# Photodynamic inactivation of antibiotic-resistant bacteria in whole blood using riboflavin photodynamic method

**DOI:** 10.3389/fmicb.2024.1404468

**Published:** 2024-07-02

**Authors:** Liguo Zhu, Changqing Li, Deqing Wang

**Affiliations:** ^1^Department of Blood Transfusion, Qilu Hospital (Qingdao), Cheeloo College of Medicine, Shandong University, Qingdao, China; ^2^Institute of Blood Transfusion, Peking Union Medical College and Chinese Academy of Medical Sciences, Chengdu, China; ^3^Department of Blood Transfusion Medicine, The First Medical Center, Chinese PLA General Hospital, Beijing, China

**Keywords:** antibiotic-resistant bacteria, bacteremia, photodynamic inactivation, riboflavin, whole blood

## Abstract

Treating bacteremia caused by antibiotic-resistant bacteria is a global concern. Antibacterial photodynamic inactivation is a promising strategy to combat it. However, it’s challenging to achieve the inactivation of antibiotic-resistant bacteria in whole blood because of its opacity and complexity. We investigated a riboflavin photodynamic method to effectively inactivate antibiotic-resistant bacteria in whole blood. Four strains of antibiotic-resistant bacteria were isolated, identified, and cultured in this research: methicillin-resistant *Staphylococcus aureus* (MRSA), pan-drug-resistant *Acinetobacter baumannii* (PDRAB), ESBLs-producing *Escherichia coli* (EPEC) and pan-drug-resistant *Klebsiella pneumoniae* (PDRKP). To simulate bacteremia, antibiotic-resistant bacteria was added into whole blood. Whole blood was treated using riboflavin photodynamic method with ultraviolet irradiation (308 nm and 365 nm). The ultraviolet irradiation dose was divided into 18 J/cm^2^, 36 J/cm^2^, and 54 J/cm^2^. Microbial count of antibiotic-resistant bacteria in whole blood was used for evaluating inactivation effectiveness. The roles of red blood cells, lymphocytes, coagulation factors, and platelets in whole blood were assessed. In results, inactivation effectiveness increased as the ultraviolet dose increased from 18 J/cm^2^ to 54 J/cm^2^. At the dose of 18 J/cm^2^, inactivation effectiveness of four antibiotic-resistant bacteria were more than 80%, while only 67% of MRSA. The antibacterial effect was enhanced by the combination of riboflavin photodynamic treatment and antibiotic. The red blood cell function was susceptible to ultraviolet dose. At the dose of 18 J/cm^2^, hemolysis rate was less than 0.8% and there was no change in levels of ATP and 2,3-DPG. At the same dose, the proliferation, cell killing, and cytokine secretion activities of lymphocytes decreased 20–70%; Factor V and Factor VIII activities decreased 50%; Fibrinogen and platelet function loss significantly but reparable. Consequently, we speculated that riboflavin photodynamic method with a ultraviolet dose of 18 J/cm^2^ was effective in inactivating four antibiotic-resistant bacteria in whole blood while whole blood function was preserved. We also provided a novel extracorporeal circulation phototherapy mode for treating bacteremia caused by antibiotic-resistant bacteria.

## Introduction

Antibiotic-resistant bacteria is a major global health concern, with new mechanisms of antibiotic-resistance are being constantly discovered (Blair et al., 2015). World Health Organization (WHO) has published a list of antibiotic-resistant “priority pathogens”—a catalogue of 12 families of bacteria (WHO, 2017). To address this issue, global scientists are constantly developing novel agents, phage therapy, and combination therapy strategies to treat infections caused by antibiotic-resistant bacteria (Paul et al., 2014; Rodvold and McConeghy, 2014; Viertel et al., 2014). However, the difficulty of treatment depends on the location of infection. In general, patients are likely to develop urinary and respiratory tract infections with antibiotic-resistant bacteria after receiving antibiotics (van Hecke et al., 2017). Additionally, bloodstream infections caused by antibiotic-resistant bacteria are common in clinical practice. For example, methicillin-resistant *Staphylococcus aureus* (MRSA) is one of the most common antibiotic-resistant pathogens responsible for bloodstream infections worldwide, with a mortality rate of 15 to 45% (Thwaites et al., 2011). Therefore, it is crucial to develop more antibiotics combination therapy strategies or other novel patterns for the treatment of antibiotic-resistant bacteria bacteremia.

Antibacterial photodynamic inactivation (PDI) is one of particularly promising antimicrobial strategies and not likely to induce antibiotic resistance in bacterial offspring. Several studies have showed that PDI is effective in inactivating antibiotic-resistant bacteria on the surface of skin and corneal (Nitzan et al., 2004; Maisch et al., 2014; Makdoumi and Bäckman, 2016; Makdoumi et al., 2017; Wong and Santiago, 2017; Leanse et al., 2020). However, there are few reports on inactivation of antibiotic-resistant bacteria in blood using PDI. Riboflavin photodynamic method, a type of PDI, has been proved to effectively inactivate viruses in plasma, bacteria in platelet concentrates, and parasites in whole blood (Ruane et al., 2004; Marschner and Goodrich, 2011; Allain et al., 2016; Jimenez-Marco et al., 2017). This is because riboflavin, as a strong endogenous photosensitizer, targets guanine bases and causes oxidative damage selectively under ultraviolet (UV) irradiation (Kumar et al., 2004; Lin et al., 2006). Several studies have showed that the presence of riboflavin enhances the level of pathogen inactivation and the rate of DNA modification in pathogen while reducing the likelihood of DNA repair compared to the use of ultraviolet irradiation alone (Kumar et al., 2004; Marschner and Goodrich, 2011; Zhu et al., 2018). Previously, our team has established a blood inactivation system using riboflavin photodynamic method and has evaluated its effectiveness in inactivating viruses in plasma and antibiotic-resistant bacteria in apheresis platelet concentrates (Zhu et al., 2015, 2018, 2020). In this research, we demonstrated for the first time that riboflavin photodynamic method is also effective in inactivating various antibiotic-resistant bacteria in whole blood. Meanwhile, we systematically investigated the impact of this inactivation treatment on various blood components in whole blood.

## Materials and methods

### Isolation and culture of antibiotic-resistant bacteria

Four strains of antibiotic-resistant bacteria, methicillin-resistant *Staphylococcus aureus* (MRSA), pan-drug-resistant *Acinetobacter baumannii* (PDRAB), ESBLs-producing *Escherichia coli* (EPEC) and pan-drug-resistant *Klebsiella pneumoniae* (PDRKP) were isolated from patients hospitalized at the Chinese PLA General Hospital. They were identified by antibiotic susceptibility test using microbial ID/AST testing system (VITEK 2, BioMerieux, France) and testing card (VITEK 2 AST-GN09, VITEK 2 AST-GN13, VITEK 2 AST-GP67, BioMerieux, France). Minimum inhibitory concentration (MIC) values in antibiotic susceptibility test were used for assessing antibiotic susceptibility. Single colony of antibiotic-resistant bacteria was isolated to be cultured on blood plate (BioMerieux, France).

### Preparation of whole blood containing with antibiotic-resistant bacteria

Whole blood units were donated by donors at the Chinese PLA General Hospital and tested as qualified. Firstly, suspensions of antibiotic-resistant bacteria were prepared by selecting a single colony of MRSA, PDRAB, EPEC, and PDRKP. Secondly, the concentration of antibiotic-resistant bacteria was measured using DESMAT meter (DenisiCHEK—plus, BioMerieux, France) and adjusted the value to 0.5 (1 × 10^8^/mL) with 0.45% saline. Thirdly, bacterial suspensions were diluted to a concentration of 1 × 10^5^/mL using 0.45% saline. Finally, whole blood was spiked with the diluted bacterial suspensions at a ratio of 1:10, resulting in a final concentration of approximate 1 × 10^4^/mL of antibiotic-resistant bacteria in whole blood. Apheresis platelet concentrates used in this research were collected from donors at the Chinese PLA General Hospital using the Automatic Blood Component Processing System (CompoMat G5, Fresenius Kabi, Germany).

### Riboflavin photodynamic inactivation process and effectiveness determination

The inactivation process was similar to our previous research (Zhu et al., 2020). Three milliliters of whole blood containing with MRSA, PDRAB, EPEC or PDRKP was injected into the small illumination bag (40 mm × 60 mm) respectively and the thickness of whole blood was approximate 1.3 mm. The illumination bag was made of special material called ethylene vinyl acetate copolymer which ultraviolet transmittance was above 99%. Then, 100 μL of riboflavin sodium phosphate (Jiangxi Pharmacy, China) with a concentration of 8,000 μmol/L was added into illumination bag to make the final concentration in whole blood was approximate 260 μmol/L. The illumination bag was placed in the blood inactivation system with 22 ± 4°C and exposed to UVA combined with UVB irradiation (irradiation distance was 5 cm). The parameters of UV lamps used in this research was the same to our previous research (Zhu et al., 2015, 2020). The wavelength of UVA fluorescent lamp (TL-D 15 W, Philips, Netherlands) was 365 nm as a peak and the wavelength of UVB fluorescent lamp (G15T8E, Sankyo Denki, Japan) was 308 nm as a peak. UVA and UVB fluorescent lamps were arranged in cross pattern, each contributing 50% irradiation. The total combined irradiation intensity was 15 mW/cm^2^ which measured directly by a UV light meter (YK-35UV, Lutron, Taiwan). The ultraviolet irradiation dose was calculated as J/cm^2^: 18 J/cm^2^ required 20 min duration, 36 J/cm^2^ required 40 min and 54 J/cm^2^ required 60 min. The experiment was divided into four groups: untreated, treated by riboflavin photodynamic method with 18 J/cm^2^, 36 J/cm^2^ and 54 J/cm^2^. Microbial viability of pathogenic bacteria was used for determining inactivation effectiveness during inactivation process. Colony-forming unit (CFU) was determined on blood plate using the plate counting method. Inactivation effectiveness was represented as “%”: [(CFU in untreated − CFU in treated)/CFU in untreated] × 100.

### Assessment of red blood cell function

Red blood cell count, hemoglobin (Hb), hematocrit (Hct) and mean corpuscular volume (MCV) in whole blood were directly measured using Automatic Blood Analyzer (XS-990i, Sysmex, Japan). The hemolysis rate of red blood cells in whole blood was measured using a free hemoglobin test kit (Nanjing Jiancheng Bioengineering Institute, China) and calculated by free Hb, Hb and Hct. The hemolysis rate was calculated by: % Hemolysis = [(1 − Hct) × Free Hb g/L × 100]/Total Hb g/L. Ions and pH were directly measured using Blood gas, Oximetry, Electrolyte and Metabolite Analyzer (ABL9, Radiometer, Denmark). Adenosine triphosphate (ATP) in red blood cells was measured using a ATP test kit (Nanjing Jiancheng Bioengineering Institute, China). 2,3-diphosphoglyceratewas (2,3-DPG) in red blood cells was measured using a 2,3-DPG test kit (Wuhan Cusabio, China).

### Assessment of lymphocyte function

White blood cell count in whole blood was measured directly using an Automatic Blood Analyzer (XS-990i, Sysmex, Japan). Lymphocytes were separated using a human peripheral blood lymphocyte isolation kit (Tianjin Haoyang Biological, China). The proliferation viability of lymphocytes was detected by cell proliferation/toxicity assay using cell counting kit-8 (Dojindo Laboratories, Japan). The cell killing activity of lymphocytes was evaluated by calculating the survival rate of Jurkat cells after co-cultured them with fluorescent labeled Jurkat cells at a ratio of 10:1. The cytokine secretion activity of lymphocytes was evaluated by measuring interleukin, tumor necrosis factor, and interferon in the supernatant using the Liquid Auspension Chip System (FlexMAP 3D, Luminex, United States) with a human high sensitivity T cell magnetic bead kit (Millipore Corporation, United States) after stimulating the lymphocytes with phytohemagglutinin (PHA) at a final concentration of 1 μg/mL.

### Assessment of plasma factor activity

Whole blood was centrifuged with a centrifuge at 3000 × G for 5 min and serum was collected to measure plasma factor activity. Fibrinogen (Fib) activity was measured by the Coagulation Analyzer (STA Compact, Diagnostica Stago, France) using the Clauss clotting method with fibrinogen kit (STA-fibrinogen 5 kit, Diagnostica Stago, France). Factor V and Factor VIII activities were measured by the IL Coagulation System (Instrumentation Laboratory Company, Bedford, United States) using the prothrombin time assay with FV-deficient plasma kit (Hemosil, IL Company, Bedford, United States) and the activated partial thromboplastin time assay with FVIII-deficient plasma kit (Hemosil, IL Company, Bedford, United States) respectively.

### Assessment of platelet function

Platelet count (PLT), mean platelet volume (MPV), platelet distribution width (PDW), in whole blood were measured directly using an Automatic Blood Analyzer (XS-990i, Sysmex, Japan). Platelet coagulation function in whole blood was directly measured using Thromboelastography Hemostasis System (TEG5000, Haemoscope Corporation, United States) with an activated coagulation test kit (Haemoscope Corporation, United States). MA value in TEG parameters was used for reflecting platelet coagulation status and the normal range was 50–70.

### Data statistical analysis

Three times were repeated in each experiment at least. The mean value and standard deviation were calculated and two-sample *t*-test was employed to analysis the difference using *SPSS* 17.0 software (*p* < 0.05 means significant difference).

## Results

### Inactivation effectiveness on four antibiotic-resistant bacteria in whole blood

**Figure 1:**
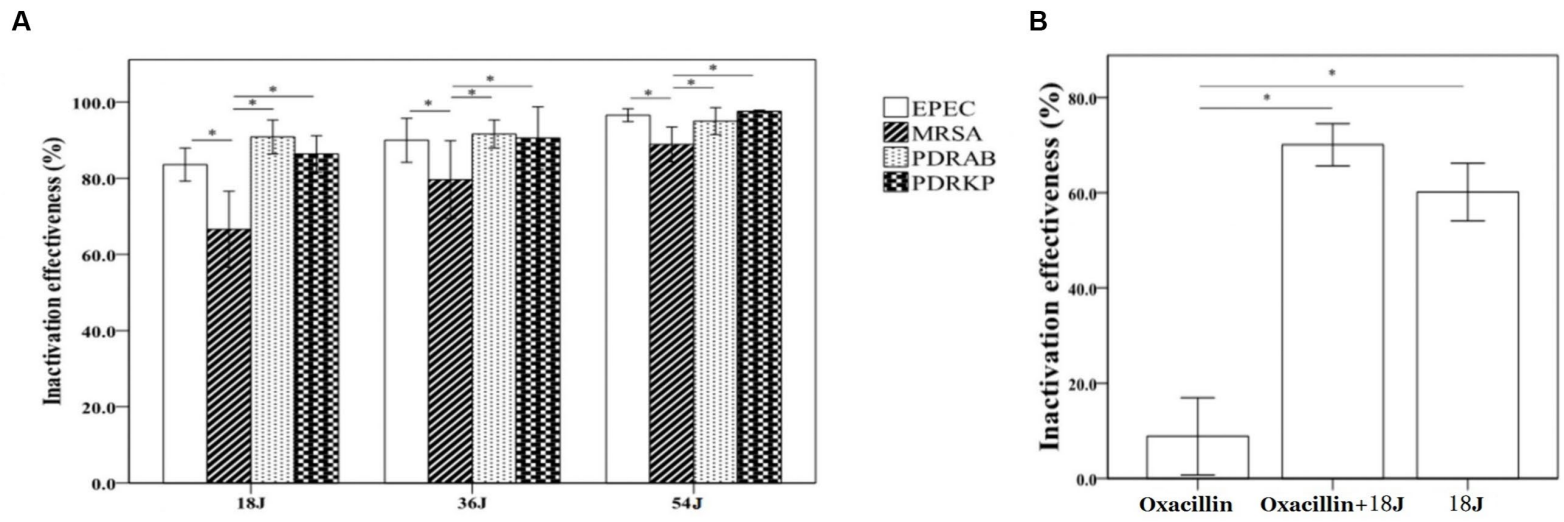
Inactivation effectiveness on four antibiotic-resistant bacteria in whole blood using riboflavin photodynamic method. The bacterial concentration of untreated control is approximate 1 × 10^4^/mL. The energy was intended per unit square cm. **(A)** The inactivation effectiveness was stronger with the increase of ultraviolet irradiation dose. At the dose of 18 J/cm^2^, inactivation rates were all above 80%, but 67% of MRSA was much lower than that of PDRAB, PDRKP or EPEC. At the highest dose of 54 J/cm^2^, the inactivation rates of four antibiotic-resistant bacteria were all above 90%. **(B)** Compared with riboflavin photodynamic treatment alone, combining treatment with oxacillin addition (final concentration of 100 ng/mL) significantly enhanced the inactivation effectiveness on MRSA. ^*^p < 0.05.

A dose-dependent relationship was observed in the inactivation effectiveness on MRSA, PDRKP, and EPEC in whole blood using riboflavin photodynamic method with different ultraviolet doses (Figure 1A). Inactivation effectiveness was higher with the increase of ultraviolet dose from 18 J/cm^2^ to 54 J/cm^2^. There was a difference in inactivation effectiveness on species of antibiotic-resistant bacteria in whole blood at the same ultraviolet dose. For example, the inactivation effectiveness on MRSA was lower than that of PDRAB, PDRKP, and EPEC. (Figure 1A). Furthermore, the inactivation effectiveness, such as MRSA, may be enhanced by the combination of riboflavin photodynamic treatment and antibiotic (Figure 1B).

### Function of red blood cells in whole blood with treatment

**Figure 2:**
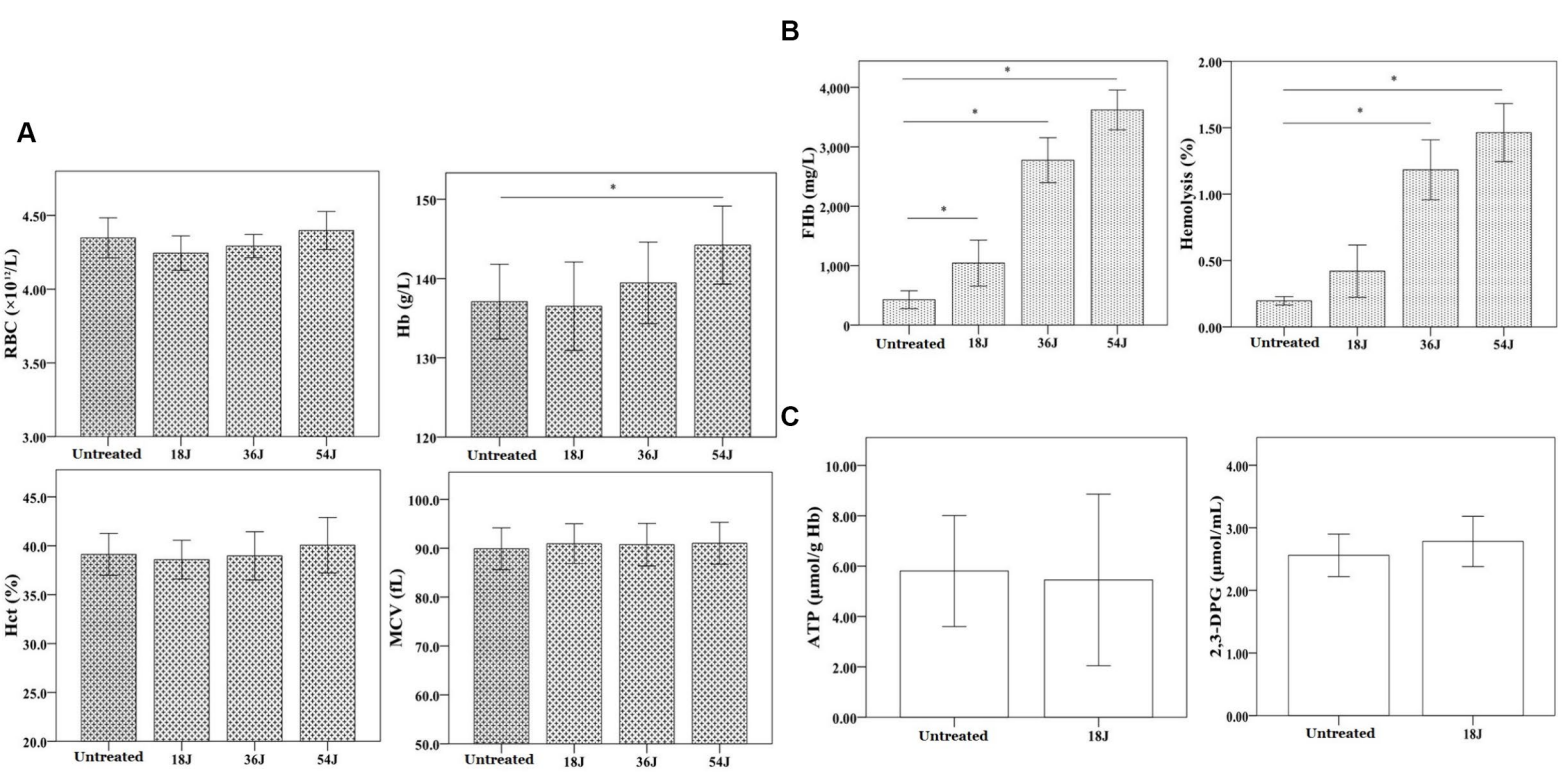
Influence on red blood cell function in whole blood. The energy was intended per unit square cm. **(A)** There was no change in the quantity, form and size of red blood cells in whole blood with different ultraviolet doses from 18 J/cm^2^ to 54 J/cm^2^. The level of Hb increased progressively as the ultraviolet dose increased. **(B)** At the dose of 18 J/cm^2^, the hemolysis rate of red blood cells was approximate 0.4%, but the hemolysis rates were all above 0.8% at the dose of 36 J/cm^2^ and 54 J/cm^2^. **(C)** At 18 J/cm^2^, the levels of ATP and 2,3-DPG in red blood cells were not significantly changed. ^*^p < 0.05.

There was no significant impact on routine parameters of red blood cells in whole blood with treatment. However, the hemolysis rate of red blood cells in whole blood increased as the ultraviolet irradiation dose increased from 18 J/cm^2^ to 54 J/cm^2^ (Figure 2). At the dose of 18 J/cm^2^, hemolysis rate of red blood cells in whole blood was less than 0.8% and there was no significant change in ATP and 2,3-DPG of red blood cells. According to a number of guidelines, hemolysis rate <0.8% was safe. Therefore, the treatment with 18 J/cm^2^ was acceptable and optimal in this results.

### Bio-metabolism in whole blood with optimal treatment

**Figure 3:**
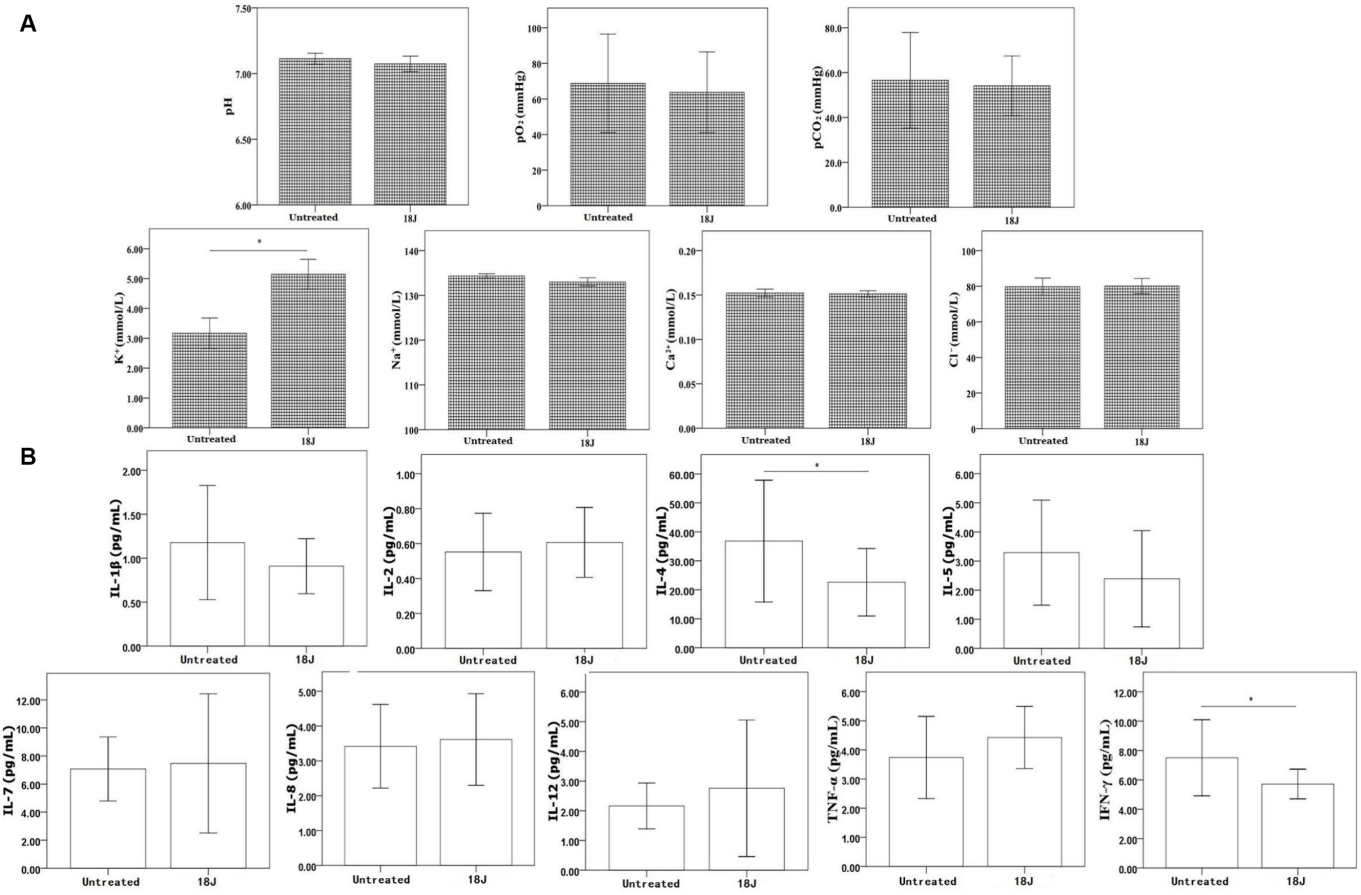
Influence on bio-metabolism in whole blood. The energy was intended per unit square cm. **(A)** Compared with untreated, the levels of pH, pO_2_, pCO_2_, Na^+^, and Cl^−^ in whole blood with optimal treatment not changed significantly, but K^+^ level increased. **(B)** For cytokines, the levels of IL-4 and IFN-γ slightly decreased in whole blood with optimal treatment, but other cytokines not changed significantly. ^*^p < 0.05.

With the optimal treatment (18 J/cm^2^), the levels of blood gas (pH, *p*CO_2_ and *p*O_2_), electrolytes (Na^+^, Ca^2+^, Cl^−^), and cytokines (IL-1β, IL-2, IL-5, IL-7, IL-8, IL-12, TNF-α, IFN-γ) in whole blood not changed considerably. However, the levels of K^+^, IL-4, and IFN-γ in whole blood with this treatment deceased significantly compared with untreated (Figure 3).

### Function of lymphocytes in whole blood with optimal treatment

**Figure 4:**
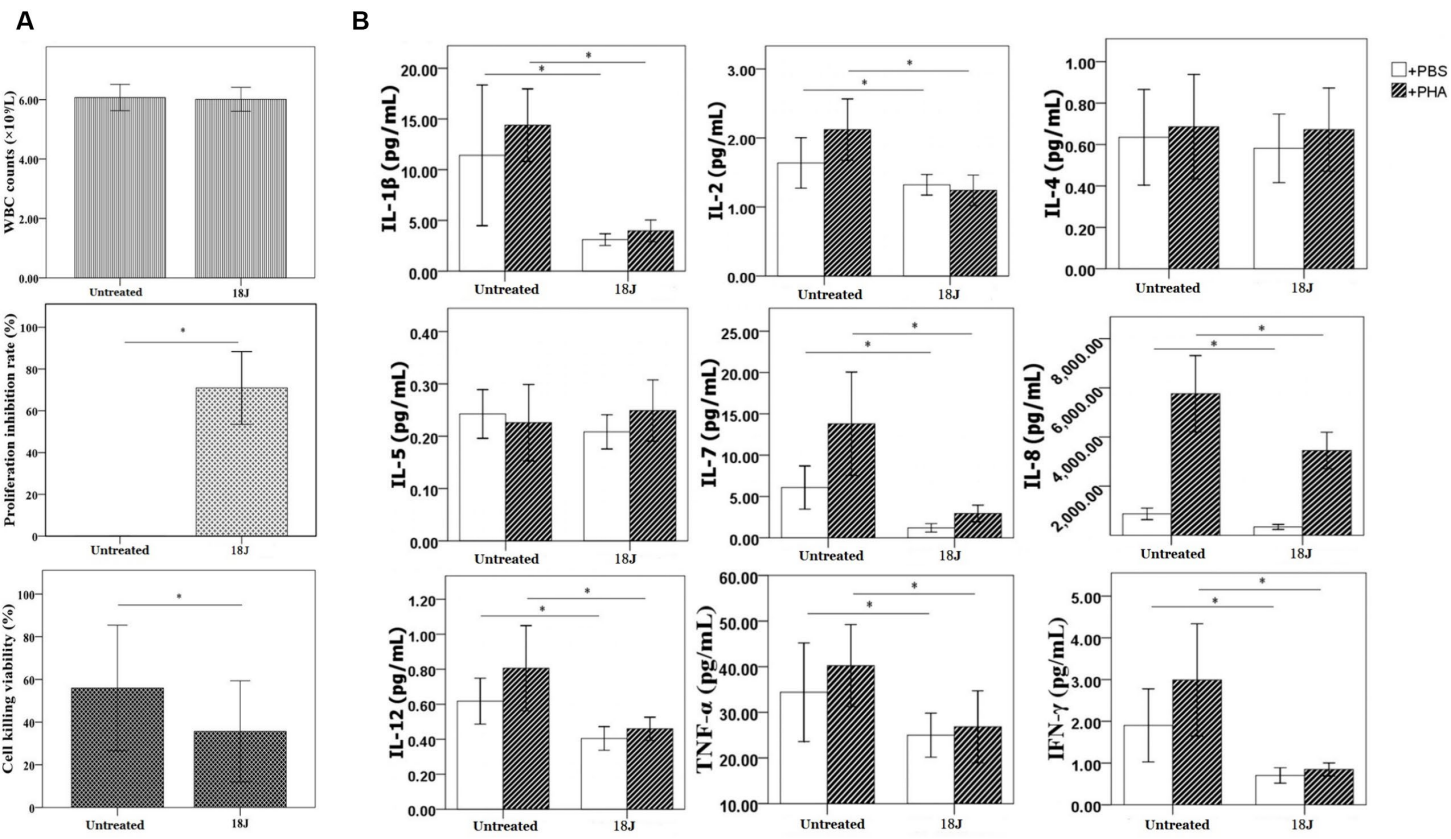
Influence on lymphocyte function in whole blood. The energy was intended per unit square cm. **(A)** With optimal treatment of 18 J/cm^2^, there was no change in the quantity of white blood cells in whole blood, but this treatment inhibited the proliferation viability of lymphocytes (proliferation inhibition rate was 70%) and cell killing viability of lymphocytes (decreased 20%) in whole blood. **(B)** Lymphocytes’ activity to secrete cytokines was definitely damaged, the levels of cytokines produced by lymphocytes decreased 50–70% with the exception of IL-4 and IL-5. ^*^p < 0.05.

With the optimal treatment (18 J/cm^2^), there was no impact on the quantity of white blood cells in whole blood, but this treatment inhibited the proliferation, cell killing and cytokine secretion activities of lymphocytes in whole blood (Figure 4). Compared with untreated, the proliferation viability was more susceptible and decreased 70%; the impact on activity to secrete cytokine was moderate; the cell killing viability was more preserved.

### Coagulation function of whole blood with optimal treatment

**Figure 5:**
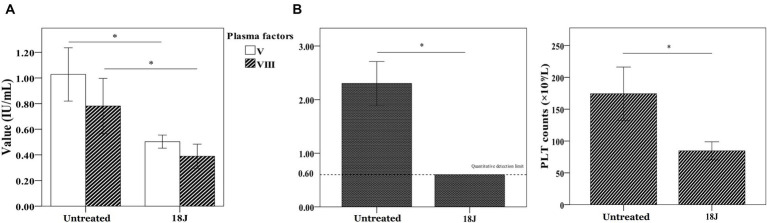
Influence on coagulation function in whole blood. The energy was intended per unit square cm. **(A)** Compared with untreated, Factor V and Factor VIII activities decreased 50% and fibrinogen loss largely. **(B)** Platelet amount in whole blood decreased 50% compared with untreated. ^*^p < 0.05.

**Figure 6:**
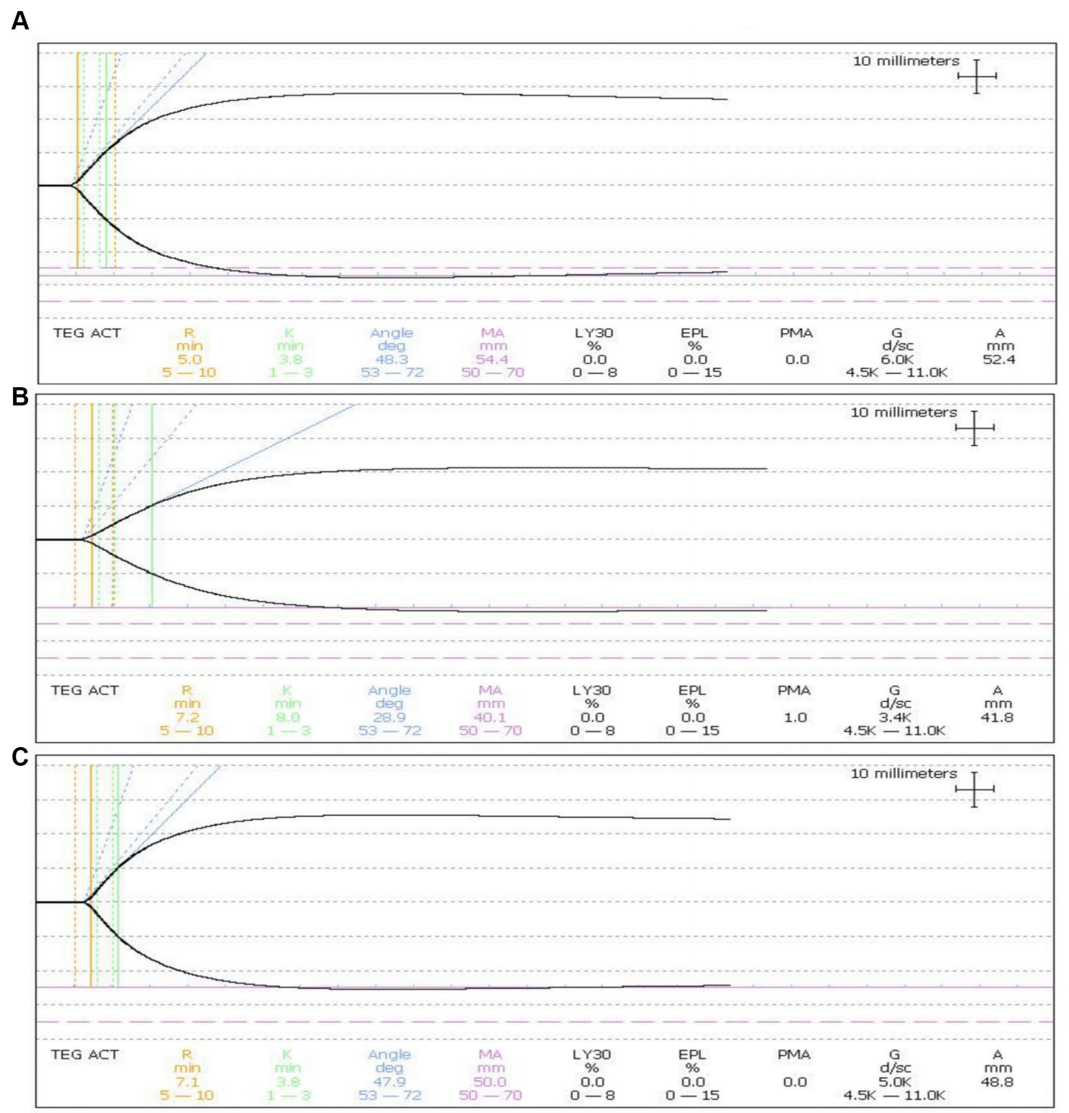
Influence on platelet function in whole blood. **(A)** The MA value of TEG was 54.4 in untreated whole blood. **(B)** MA value dropped to 40.1 in treated whole blood. **(C)** MA value rebounded to 50.0 in treated whole blood with the apheresis platelets addition at a ratio of 1:10.

A significant damage to coagulation function of whole blood with optimal treatment was observed. Factor V and Factor VIII, and fibrinogen loss largely. Platelet amount decreased 60% (Figure 5). With the optimal treatment (18 J/cm^2^), the MA value in TEG results of whole blood dropped from 54 to 40 (Figure 6). However, with apheresis platelets addition (1:10) in whole blood, MA value rebounded to 50.0.

## Discussion

In a variety of contexts, including food, medicine, and even water disinfection, PDI of antibiotic-resistant bacteria has been researched (Almeida et al., 2014; Maisch et al., 2014; Makdoumi and Bäckman, 2016; Makdoumi et al., 2017; Ahmed et al., 2020). However, it is challenging to achieve the inactivation of antibiotic-resistant bacteria in blood because of light source penetration and photosensitizer safety. We have conducted extensive research to deal with this issue using riboflavin photodynamic method.

Numerous species of antibiotic-resistant bacteria were likely to cause refractory bacteremia. Four strains of antibiotic-resistant bacteria were chosen as typical samples for this investigation. MRSA, a gram-positive antibiotic-resistant bacteria, was a significant contributing factor to bloodstream infections that were highly fatal (Moore et al., 2012). PDRAB, a gram-negative antibiotic-resistant bacteria, exhibited an extreme antimicrobial resistance profile, high rate of antibiotic resistance, and abysmal outcomes (up to 70% mortality rate) (Wong et al., 2017; Hu et al., 2018). EPEC, a gram-negative antibiotic-resistant bacteria, exhibited a high prevalence and continued rise in bloodstream infections resulting in limited therapy options (Tamma and Rodriguez-Bano, 2017; Bonomo et al., 2018). PDRKP, a gram-negative antibiotic-resistant bacteria, showed a noticeable increase and emerged as a serious clinical and public health concern over the past decade (Wyres et al., 2020; Zhang et al., 2020).

Since whole blood was complex and opaque, it would require more irradiation dose and thinner blood to achieve effective inactivation of antibiotic-resistant bacteria in whole blood. In this research, our blood inactivation system using riboflavin photodynamic method showed an effective inactivation effect on MRSA, PDRAB, EPEC and PDRKP in whole blood (Figure 1). The inactivation effectiveness was stronger with the increase of ultraviolet irradiation dose. However, there was a difference in inactivation effectiveness on species of antibiotic-resistant bacteria in whole blood at the same ultraviolet dose. At the lowest dose of 18 J/cm^2^, inactivation rates were all above 80%, but 67% of MRSA was much lower than that of PDRAB, PDRKP or EPEC. At the highest dose of 54 J/cm^2^, the inactivation rates of four antibiotic-resistant bacteria were all above 90%. This results indicated that MRSA in whole blood was less susceptible to riboflavin photodynamic treatment than other gram-negative antibiotic-resistant bacteria. Previous research also indicated that different strains of bacteria exhibited varying sensitivities to various PDI (Taha et al., 2017; Ziganshyna et al., 2020). Indeed, ultraviolet irradiation alone had a certain antibacterial effect, but riboflavin combined with ultraviolet would improve this effect and not induce resistance (Kumar et al., 2004; Marschner and Goodrich, 2011). The MIC values of four residual antibiotic-resistant bacteria in whole blood were not altered after inactivation treatment. This outcome was consistent with other earlier research: PDI did not lead to development of antibiotic resistance (Al-Mutairi et al., 2018). Furthermore, our research showed that inactivation effect against antibiotic-resistant bacteria, such as MRSA, may be enhanced by the combination of riboflavin photodynamic treatment and antibiotic. It was highlighted that the initial concentration of antibiotic-resistant bacteria in whole blood in this research was 1 × 10^4^ CFU/mL, which was far greater than what was often found in clinical settings. The contaminated concentration for bloodstream bacterial infection was 1–2 CFU/mL in adults and 10–100 CFU/mL in children (Cantey and Milstone, 2015; Akova, 2016; Kern and Rieg, 2020). Therefore, this research was only a preliminary experiment to explore the scientific possibility of photodynamic inactivation against antibiotic-resistant bacteria in whole blood. Whether the inactivation rate of 80, 90% or even 99% has clinical significance, it is not completely clear and it will require more clinical trials to confirm the efficacy shown in this research.

It was important to take into account whole blood function while inactivating antibiotic-resistant bacteria in whole blood. The first factor was the hemolysis rate of red blood cells in whole blood. According to a number of guidelines, hemolysis rate of red blood cells in treated or washed blood for clinical use should be less than 0.8% (Sivertsen et al., 2020; Kumukova et al., 2021). In this research, the rate of hemolysis increased as the ultraviolet irradiation dose increased (Figure 2). Therefore, it was determined that the optimal treatment dosage was 18 J/cm^2^. Following treatment with the optimal dose, hemolysis rate of red blood cells in whole blood was less than 0.8%, and there was no significant change in ATP and 2,3-DPG of red blood cells. The levels of blood gas, oximetry, electrolyte, and cytokine concentration in whole blood not changed considerably, with the exception of K^+^, IL-4, and IFN-γ (Figure 3). The increase of K^+^ was probably related to red blood cell hemolysis. It will require more research to determine the safe range for hemolysis rate in peripheral blood.

The viability of cytokine secretion, cell killing, and proliferation were among the roles played by lymphocytes in whole blood. Since lymphocytes were nucleated cells, riboflavin photodynamic method was certain to inactivate some lymphocytes whether in red blood cell suspensions or whole blood (Marschner et al., 2010). Finding the balance point between inactivating antibiotic-resistant bacteria and maintaining whole blood function was crucial. In this research, the optimal treatment obviously inhibited the proliferation activity of lymphocytes in whole blood, but not totally deactivated (Figure 4). This treatment also evidently decreased, albeit not entirely eliminated, cell killing and cytokine secretion viabilities of lymphocytes in whole blood. Lymphocytes with function retention of 50% whether can still play a certain immune role *in vivo*, it will require further research.

Riboflavin photodynamic method may negatively impact the coagulation function of treated whole blood because of ultraviolet penetration and reactive oxygen species generation (Feys et al., 2014; Schubert et al., 2015). UVB used in this research was mainly responsible for this phenomenon due to penetration of UVB to destroy protein structure. This research found that fibrinogen in treated whole blood showed considerable damage, Factor V and Factor VIII showed less damage than fibrinogen (Figure 5). TEG, an alternative technology, was able to accurately reflect coagulation activity of platelets in clinical applications (Whiting and DiNardo, 2014). In this research, platelet count and TEG value in treated whole blood exhibited slight decrease compared to untreated whole blood (Figure 6). Nevertheless, coagulation status can be corrected and returned to normal range by adding 10% apheresis platelets into treated whole blood. In other words, the addition of 10% apheresis platelets in this investigation was equivalent to transfusion of two doses of apheresis platelets into an adult. Overall, despite the adverse consequences of this treatment, it is reparable.

**Figure 7:**
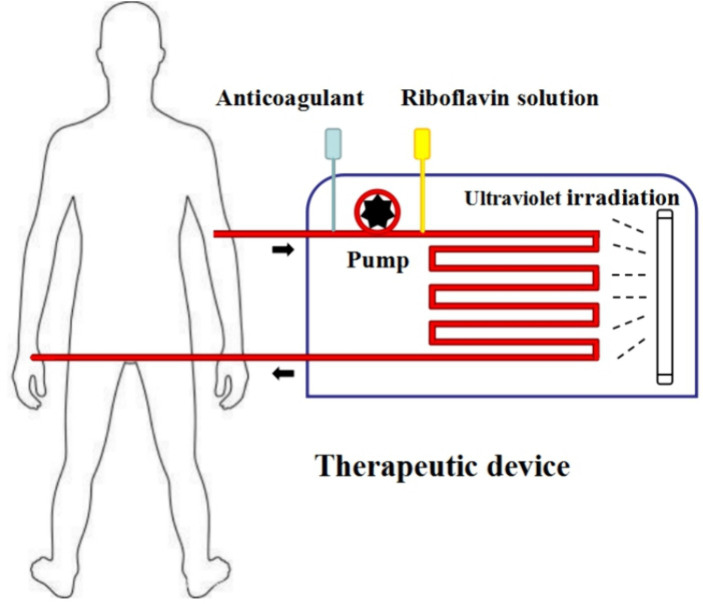
Phototherapy mode prospect. Step1: whole blood from patients is collected into therapeutic device via a peristaltic pump. Step2: riboflavin solution is added into whole blood with a certain concentration. Step3: whole blood mixing with riboflavin solution flows through the ultraviolet irradiation area with a certain speed. Step4: whole blood with irradiation is re-transfused into patient’s peripheral blood.

In the future, more phototherapy patterns may be developed based on a blood recirculating system using riboflavin photodynamic method (Zhu et al., 2015; Kim et al., 2018). We also designed a novel phototherapy mode using riboflavin photodynamic method for treating patients with antibiotic-resistant bacteria bacteremia: it comprises of whole blood collection, riboflavin addition, photoinactivation, and reinfusion technology in a single closed system (Figure 7). It is anticipated that one or more extracorporeal circulation phototherapy process will achieve a considerable reduction of antibiotic-resistant bacteria load in patient’s peripheral blood. As a supplementary treatment mode, it is expected to delay infectious disease progression by reducing drug-resistant bacteria load in circulating blood. It should be noted that coagulation function decline may be one of the main adverse effects. In order to mitigate adverse effects associated with the treatment, apheresis platelets, plasma or coagulation medication is appropriately administered to improve coagulation in accordance with the patient’s coagulation state following phototherapy treatment.

Naturally, there were some limitations in this experimental research. In this research, the whole blood for irradiation was in illumination bag but not flowing and the capacity was small. This research was performed in an exploratory mode to demonstrate the possibility of photodynamic inactivation on antibiotic-resistant bacteria in whole blood using riboflavin photodynamic method, rather than in a final clinical treatment mode. In the following research, we will establish a circulating irradiation device to verify the effectiveness and optimize the parameters via large volume whole blood. We expect less hemolysis and whole blood function damage in circulating treatment compared with treatment in a bag. Additionally, whether phototherapy for bacteremia is clinically effective or not, it will also require more clinical trials in the future.

## Conclusion

This is the first research using riboflavin photodynamic method against antibiotic-resistant bacteria in whole blood. At dose of 18 J/cm^2^, this photodynamic treatment was effective in inactivating four antibiotic-resistant bacteria in whole blood while whole blood function was partially damaged. According to the exploratory results of this research, a novel extracorporeal circulation phototherapy mode for treating patients with bacteremia caused by antibiotic-resistant bacteria will be feasible in the future.

## Data availability statement

The original contributions presented in the study are included in the article/supplementary material, further inquiries can be directed to the corresponding author.

## Ethics statement

The studies involving humans were approved by the Ethics Committee of Qilu Hospital (Qingdao), Cheeloo College of Medicine, Shandong University (No. KYLL-KS-2021165). The studies were conducted in accordance with the local legislation and institutional requirements. The human samples used in this study were acquired from primarily isolated as part of your previous study for which ethical approval was obtained. Written informed consent for participation was not required from the participants or the participants’ legal guardians/next of kin in accordance with the national legislation and institutional requirements.

## Author contributions

LZ: Writing – original draft, Visualization, Validation, Software, Project administration, Methodology, Investigation, Funding acquisition, Formal analysis, Data curation, Conceptualization. CL: Writing – review & editing, Supervision. DW: Writing – review & editing, Supervision, Resources, Conceptualization.
